# Clearing the air: protocol for a systematic meta-narrative review on the harms and benefits of e-cigarettes and vapour devices

**DOI:** 10.1186/s13643-016-0264-y

**Published:** 2016-05-21

**Authors:** Marjorie MacDonald, Renee O’Leary, Tim Stockwell, Dan Reist, on behalf of the Clearing the Air project team

**Affiliations:** School of Nursing, University of Victoria, Room A402a, HSD Building, 3800 Finnerty Road, Victoria, BC V8P 5C2 Canada; Centre for Addictions Research of British Columbia, University of Victoria, Victoria, BC V8W 2Y2 Canada

**Keywords:** E-cigarettes, Vapour devices, Smoking cessation, Harm reduction, Meta-narrative synthesis, Meta-narrative review, Systematic review, Health impacts

## Abstract

**Background:**

Under the shadow of the tobacco epidemic, the sale and use of e-cigarettes and other vapour devices is increasing dramatically. A contentious debate has risen within public health over the harms and benefits of these devices. Clearing the Air seeks to clarify the issues with a systematic review that informs the pressing regulatory and public health decisions to be made regarding these new products.

**Methods/design:**

Using an integrated knowledge translation approach, public health researchers and knowledge users will work collaboratively throughout the project. Our research questions are the following: (1) What are the health risks and benefits of vapour devices, and how do these compare to cigarettes? (2) What is the harm reduction potential of vapour devices for individuals, the environment, and society? (3) Does youth vapour device experimentation lead to cigarette use? (4) Can vapour devices be effective aids for tobacco cessation? and (5) What is the potential toxicity of second-hand vapour?

We are using meta-narrative review to synthesize studies from diverse research traditions because of its capacity to address contestations around a topic. The project has six phases. In the planning phase, we finalized the research questions. In the search phase, we are locating academic publications and grey literature aided by a research librarian. The mapping phase involves categorizing these papers into research traditions to understand different perspectives on the evidence for each research question. In the appraisal phase, we will select and evaluate the relevant papers. Finally, in the synthesis phase, using analytic techniques unique to meta-narrative methodology, we will compare and contrast the evidence from different research traditions to answer our research questions, identifying overarching meta-narratives. In the final stage, the full team will draft recommendations to be disseminated through a variety of knowledge translation strategies.

**Discussion:**

Meta-narrative synthesis has the unique capacity to expose the debates that are influencing the interpretation of empirical studies on vapour devices. We seek to “clear the air” with an even-handed review of the evidence and an understanding of the tensions within public health so that we can offer clear-headed recommendations for policy, regulation, and future research.

**Systematic review registration:**

PROSPERO CRD42015025267

**Electronic supplementary material:**

The online version of this article (doi:10.1186/s13643-016-0264-y) contains supplementary material, which is available to authorized users.

## Background

The tobacco epidemic causes almost 6 million deaths worldwide every year and, if current trends are unchecked, the rate will rise to 8 million by 2030 [1]. At the same time, the sale and use of electronic cigarettes and vapour devices is increasing dramatically, from US $573 million in 2009 to US $6.48 billion in 2014 [2]. The public health community is divided, even polarized, over how the use of these devices will impact the tobacco epidemic [3]. Some tobacco control advocates predict that e-cigarettes will increase rates of cigarette uptake [4, 5], especially among youth [6, 7]. Others envision that these devices have potential for aiding cessation efforts, or reducing harm among people who continue to smoke [8–11].

In Canada, in 2013, a group of 90 public health and addiction specialists participated in a forum on the potential responses to the growing use of vapour devices [12]. Although consensus was achieved on the need for product control and regulating sales to minors, dissent remained on all other issues. The debate in the public health community is fueled by the lack of a comprehensive review of the research on the range of health and social impacts of these devices. At present, some research suggests benefits for the use of vapour devices [13–15], while other evidence identifies harms [16–19]. Even the conclusions of systematic reviews are contradictory, with some claiming minimal harm and with others not recommending their use [9, 20–26]. Resolving these contradictions is essential to move forward on policy and regulation.

In this paper, we use the term *vapour devices* to include the wide range of consumer products that heat nicotine and non-nicotine liquids to produce vapour that is inhaled by the user. The various product designs and terms include electronic or e-cigarette, electronic nicotine delivery system (ENDS), cig-a-like, mod, e-hookah, vape pen, tank system, vaping device, e-cigar, and third generation devices.

Both the public and the public health community have an interest in the potential health consequences from vapour device use. Individuals are worried about possible harm to themselves from their exposure to vapour. For example, the absence of regulation on legal non-nicotine devices in Canada has lead to vapour devices being used where cigarette use is prohibited. As a consequence, people in schools, workplaces, and public venues are concerned about the potential harms to bystanders exposed to possible aerosol contaminants (i.e. second-hand vapour). From a public health perspective, in addition to questions about potential health risks, these devices raise issues about their potential contribution to health inequities. Even though smoking is declining in the overall population, sexual minorities, street-involved people, persons with mental illness, and indigenous populations all have much higher rates of smoking [27–29]. If vapour device use results in additional harms, it may disproportionately affect these populations. Conversely, if these devices have harm reduction potential or aid in cessation, they may substantially benefit health among these groups.

### Purpose and research questions

The purpose of this meta-narrative review is to synthesize the available evidence on vapour devices with respect to health and social impacts and their harm reduction potential. We are funded by the Canadian Institutes of Health Research through a peer-reviewed Knowledge Synthesis grant. Because we use only secondary data in the form of published materials and articles from the grey literature, no human subject ethical approval is necessary. Our approach uses an integrated knowledge translation model [30] in which public health researchers and knowledge users (e.g. policy and decision makers) work collaboratively to identify the research questions, interpret the results, make recommendations, and engage in knowledge translation.

In our initial scoping of the literature in April 2014, we located 101 primary research studies exploring a range of topics related to these devices including use patterns (26), health impacts on users and by-standers (24), prevalence (15), use in tobacco cessation (13), product toxicology (10), youth uptake (9), and consumer marketing (4). We noted a steadily increasing number of studies on vapour devices every year since 2009, as has been reported in a bibliometric analysis conducted in June, 2014 [31]. This sets the stage for a broad-based systematic review.

The research questions, developed in consultation with our knowledge user partners, are:

1. What are the health risks and benefits of vapour devices, and how do these compare to cigarettes?
2. What is the harm reduction potential of vapour devices for individuals, the environment, and society?
3. Does youth vapour device experimentation lead to cigarette use?
4. Can vapour devices be effective tools for tobacco cessation?
5. What is the potential toxicity of second-hand vapour?

The goal of *Clearing the Air: A Systematic Meta-Narrative Review on the Harms and Benefits of E-Cigarettes and Vapour Devices* is to generate a research synthesis that can inform the pressing regulatory and public health decisions to be made, specifically in British Columbia and Canada but also internationally. We seek to “clear the air” in this contentious public health debate with an appeal to the available evidence. This protocol paper describes how the research will be conducted.

## Methods/design

We selected meta-narrative synthesis (or review), as the most appropriate approach for the study. As noted above, studies on vapour devices have been conducted in diverse research traditions, with widely different methodologies, and often different, although related, research questions. This heterogeneity makes it difficult to apply a more traditional systematic review approach because each of the different research traditions may only address one or perhaps two of our research questions. In addition, our preliminary review revealed that the same evidence was often used in support of completely contradictory conclusions.

A meta-narrative review is a relatively recent constructivist synthesis methodology developed to summarize, synthesize, and interpret a diverse body of literature from multiple traditions that use different methods, theoretical perspectives, and data types [32–34]. It is systematic in that it is conducted “according to an explicit, rigorous and transparent method” ([33], p. 418). This methodology involves the judicious combination of qualitative and quantitative research evidence, the theoretical literature, and other relevant sources of data (e.g. editorials, and news items in academic journals). Meta-narrative reviewers attempt to make sense of the complex and often contested literature in a topic area. Thus, the contentious debate around vapour devices makes meta-narrative synthesis a good fit for this study because the methodology is “particularly suited to topics where there is dissent about the nature of what is being studied, and what is the best empirical approach to studying it. . . [ It can] help build common ground between social researchers and policy teams” ([35], p. 2).

The protocol described in this paper has been registered with PROSPERO [CRD42015025267]. We have included a populated PRISMA-P checklist describing our adherence to these guidelines (see Additional file 1). Because meta-narrative review is a relatively new synthesis methodology that differs in some ways from a more traditional systematic review, our protocol does not comply fully with the PRISMA-P checklist. Nonetheless, it is substantially compliant.

A defining feature of meta-narrative synthesis is its six guiding principles, which are integrated into each step of the review process. In Table 1, we identify the six principles, define them, and provide an example of how we are applying each principle in our review.

**Table 1:** Principles of meta-narrative review with application examples

Principle	Definition	Application in Clearing the Air review
Pragmatism	The reviewer is guided by the needs of the intended audience and by what is most likely to promote sense making.	Knowledge user partners approached the research team with questions about the available evidence on vapour devices, initiating the study. They were actively involved in defining the research questions, and will be involved throughout the process as the results emerge. The pragmatic need to answer these questions will include careful attention to evaluating the evidence, within the larger methodological goal of constructing overarching narratives and addressing the conflicts.
Pluralism	The topic is explored from multiple perspectives and the quality of the research or evidence is judged by criteria intrinsic to the research tradition from which it emerged. The aim is “to expose the tensions, map the diversity and communicate the complexity of how the different traditions contribute to understanding of the problem” ([32], p. 427)	Preliminary mapping suggested several research traditions each taking a different focus on the topic. Strong contestation is occurring within and between traditions on the harms and benefits of vapour devices. Team members come from different disciplinary backgrounds bringing different paradigmatic lenses to understanding the evidence. Use of evaluation criteria specific to each paradigm contributes to a pluralistic view of the evidence.
Historicity	The review explores the various research traditions as they unfold over time, including major events, key scientists, and discoveries that have shaped the tradition. The result is an emerging storyline that is not so much a “unified voice” but the unfolding of current agreements and disagreements.	The emergence of key claims and counter claims about vapour devices within specific traditions and the evidence to support those claims is being explored. Key reviews, editorials, and news items that have fueled the debate about vapour devices are being explored. This helps to contextualize how the evidence itself is understood, interpreted, and taken up within distinct traditions.
Contestation	The conflicting data from within and between research traditions are analyzed. Meta-narrative reviewers “explicitly seek to expose and unpack the ‘incommensurabilities’ that underpin conflicting data.” ([32], p. 428)	In the search and selection phase of the review, contestation was explicitly sought, with conflicting evidence and perspectives retained for review. In the synthesis phase, the goal is to find epistemological, pragmatic explanations for conflicting findings or recommendations.
Reflexivity	The reviewers must continually reflect, individually and as a team, on the emerging findings throughout the review.	We aim to cultivate in our team a spirit of critical reflexivity in which we challenge our own and each other’s assumptions and interpretations. The diversity in the team members’ backgrounds should help to promote reflection and cross-disciplinary analysis.
Peer review	The emerging findings are tested by presenting them to external reviewers in a formative way, and this feedback informs subsequent reflection and analysis	We met with our knowledge user partners to report search and preliminary mapping progress and obtained their feedback. Potential value conflicts driving the debate is providing guidance for reflection and further analysis as the review progresses. At various points in the process, knowledge users will take the findings to colleagues in their organizations to discuss and obtain feedback.

Drawn from Greenhalgh et al. [32], Greenhalgh and Wong [37], and Greenhalgh et al. [34]

The meta-narrative review quality standards [36] and training materials [37] guided the development of the research design. This project is segmented into six stages, each one incorporating a knowledge translation component. Meta-narrative synthesis methodology is not linear, and different processes may happen during one step, or may be repeated later in the review [36].

### Planning phase

The first phase is the planning stage, which has been completed. The principal investigators and research coordinator developed the draft research questions based on the questions originally posed by knowledge users. These were sent to team members including knowledge users for their review and consideration. They were asked to add additional questions that were relevant for their information needs. At the same time, we familiarized team members with the topic through the distribution of public health reviews [38–40], public opinion websites [41, 42], regulations [43, 44], and prevalence data [45, 46]. The full team reviewed, revised, and finalized the research questions at a research team meeting and during interviews with the knowledge users. In addition, each team member approved the overall approach, and chose their roles and responsibilities.

### Search phase

The second phase is the literature search process. This started with the researchers forming a mental overview of the topic through browsing the literature, discussions with colleagues, and email inquiries to experts [32]. The process was facilitated by the research coordinator who compiled a literature overview on vapour devices in 2014, and is continuing research on vapour devices for her doctoral dissertation. The entire project team has been informally networking with colleagues to inquire about studies, conference presentations, and grey literature, and in addition, several team members have subscribed to several relevant list serves with postings circulated among the whole team.

A formal literature search strategy was developed by the research coordinator, and then revised in consultation with a research librarian to include additional vapour device search terms, search fields, and databases. The search was conducted on April 14, 2015 covering the period of 2007–April 1, 2015 in 15 databases. The date of 2007 was selected as the start date for the search because that was the year in which the first published academic article on e-cigarettes appeared [47]. As specified in the training materials and quality standards for meta-narrative reviews [36, 37], the search strategy had no exclusion criteria by methodological hierarchy or technical checklist because vapour devices are a new product without an extensive literature in any tradition. We initially included publications in any language given that team members and organizational staff members speak several languages including Spanish, French, German, and Dutch in addition to English. In our search, we included all types of articles—research studies, case studies, short reports, editorials, letters, news items, and both systematic and non-systematic reviews. The non-research publications are important for mapping the development of the various research traditions and for understanding the controversies and debates about vapour devices and how the evidence has been interpreted and taken up Greenhalgh et al.’s original meta-narrative study [32, 33] included editorials, opinion articles, and non-systematic reviews for similar reasons. We also included conference abstracts and posters as potential contributors to the research traditions, but excluded dissertations because they are not yet part of the larger public health discourse. The search strategy with search terms and databases is reported in Additional file 2.

The search yielded an unexpectedly large number of publications given the recent appearance of the devices in the market. We found just over 1000 articles, compared to the 488 total publications found in the preliminary 2014 search and the 356 articles included in the Zyoud et al. [31] 2014 bibliometric analysis. Publications not referencing vapour devices in the title or abstract were given a full paper review, and excluded if vapour devices were mentioned only in passing and not a topic of discussion, for example, introductions to special issues, or requests for research. Due to the length of the search and screening processes, we were concerned that important new publications would be missed. Therefore, the same search was repeated verbatim on October 2, 2015 for the period from the end date of the last search to October 2nd, 2015. This resulted in an additional 354 publications. We are confident that we have located all relevant research studies published in this date range because the reference lists of the 24 systematic reviews from the search yielded no additional articles, only two conference abstracts.

Additional steps will be conducted for the search process. Key journals, including *Tobacco Control*, *Nicotine and Tobacco Research*, and *Harm Reduction Journal*, will be hand-searched to verify that no articles have been missed. Google citation metrics were located for all articles from the initial search. Citation metrics for articles from the updated search will be located 6 months after publication to allow the opportunity for citation. Reference tracking and citation chasing will be pilot-tested, and if fruitful, will be performed on these selected studies. Following the academic literature search, grey literature will be located through a review of 23 websites of major health and tobacco-related NGOs. Later, in keeping with the iterative design of meta-narrative review, the emerging storylines in the various research traditions will guide more focused searching to refine and flesh out areas of inquiry.

### Mapping phase

In this phase, which comprises a large part of the analytic process, the initial list of papers will be mapped by the researchers into various categories to gain a general sense of what is known about the topic areas reflected in our research questions. Papers were initially categorized by general topic areas, but in this phase, we need to define and develop the research traditions before we can map them accordingly. A preliminary list of potential research traditions is listed in Table 2. A research tradition or a meta-narrative is the unit of analysis in meta-narrative synthesis; it is defined as “a coherent theoretical discourse and a linked body of empirical research in which successive studies are influenced by preceding inquiries” ([33], p. 38). To define the various research traditions, we will be guided by the following five questions ([33], p. 28):

**Table 2:** Initial research traditions

Discipline/tradition	Areas of research and evidence
Addiction	Addictiveness, dual use, progression to tobacco use (“gateway’)
Cessation	Cessation trials, unassisted cessation, reduction, population quit rates
Clinical practice	Position statements, clinician education, surveys of clinicians
Cytotoxicology	Testing of vapour and liquids
Environmental toxicology	Second-hand vapour testing, impacts on ecology (waste products)
Epidemiology	Prevalence
Industry	Advertising and trade practices, Big Tobacco, structure (market, internet sales, vape shops), products (invention, development)
Law	Briefings on regulation, regulatory status, regulatory process
Physiology	Effects of use on the body, effects of second-hand exposure, nicotine delivery
Poison control	Accidental and intentional poisoning
Product safety	Device safety evaluation, liquid contamination, nicotine content, labeling
Sociology	User beliefs and behaviours, public opinion
Tobacco control	Regulation as tobacco, impacts on tobacco control (“renormalization”), intra-disciplinary contestation
Toxicology	Vapour composition

1. What are the parameters of the tradition—i.e. scope, historical roots, key concepts and assumptions, theoretical basis?
2. What research questions and in what priority have scientists in this tradition asked about the topic area?
3. What are the main empirical findings of relevance from the ‘quality’ literature in this tradition?
4. How has the tradition unfolded over time?
5. What are the strengths and limitations of this tradition?

It is important to keep in mind that the meta-narrative process is iterative and not linear, so there will be some back and forth between the phases, with some earlier components revisited after components later in the process are finished. For example, to answer the third question above, some aspects of the appraisal phase will need to be initiated before a tradition can be fully defined or the strengths and limitations can be identified. In effect, some revision to the research traditions may occur as we move through the process.

Next, studies are reviewed to determine the main concepts, theories, methods, questions, and instruments that characterize each research tradition [36] as well as the empirical findings, discrepant conclusions, overall strengths and limitations, and the contributions to the research question offered by each research tradition. Once mapping is completed, a team meeting will present the knowledge users with a summary of the mapping. The full team will reflect on and discuss the results and their preliminary implications for policy and public health, with the caveat that the quality appraisals and synthesis have not yet been performed. These preliminary results are being offered because the knowledge users have a pressing need for evidence for pending legislation. Following this meeting, each knowledge user, with the support of a team researcher, will present these findings to the appropriate people in their organizations to obtain feedback on the interpretation of data from those working with issues around vapour devices. The intent is to obtain ideas for how the data can be used, solicit emerging questions, and determine preferences for knowledge translation products.

### Appraisal phase

In this fourth stage, relevant papers are selected that will be brought forward for synthesis. In the mapping phase, all papers are categorized and analysed for their contribution to the development of a particular research tradition. In the appraisal phase, however, relevant papers are appraised for quality to determine the extent to which they will influence the overall conclusions. Because meta-narrative review is an interpretive and constructivist process about making sense of a body of literature rather than a technical process of categorizing data according to a checklist, we will need to select and combine data judiciously from primary sources “to produce an account of how a research tradition unfolded and why…” ([37], p. 19) as well as the major findings of that tradition. In meta-narrative synthesis, the quality criteria are drawn from the specific research traditions. Greenhalgh et al. [32] found in their extensive meta-narrative review that scientists in 13 very different research traditions tended to use similar criteria to assess the quality of studies having comparable designs. Greenhalgh et al. therefore developed a set of quality criteria for each of several study designs including experimental (randomized and non-randomized controlled trials), quasi-experimental, attribution studies, questionnaire surveys, qualitative studies, mixed methodology case studies (and other complex designs), and real-world implementation studies. We will use, and adapt as necessary, these quality checklists.

Two reviewers will rate each study, with conflicts to be resolved by the principal investigators. If appropriate, we will calculate inter-rater reliability scores. However, in one meta-narrative review conducted by the developers of the methodology [34], the authors found that in a highly constructivist process like meta-narrative review, collegial and ongoing dialogue was central to accommodating the iterative revision of interpretations. In this case, inter-rater reliability makes no sense. Because the total number of papers in our review is likely to be much smaller than in other meta-narrative reviews, there may be less diversity in interpretations. If so, it may be possible to calculate inter-rater reliability. Once the papers are assessed against the quality criteria, the researchers will classify papers as being (1) outstanding, (2) having some limitations, or (3) having many important limitations. Papers are also rated for relevance as (1) being essential, (2) to include, or (3) of marginal relevance [33]. Detailed data extraction sheets will be developed, building on the review of studies in the mapping phase, to identify important data from the study, including its quality, empirical findings, interpretations, conclusions and recommendations. The results of the appraisal phase will also be presented to the full team and discussed.

### Synthesis phase

The fifth stage is the synthesis phase. Because of the diversity of the studies and the range of our research questions, we may not be able to develop a single conceptual framework to explain the findings. Rather, particular research traditions will address only one or perhaps two of our research questions, necessitating multiple syntheses within and across traditions. To the extent possible, however, we will attempt to construct one or more overarching narratives. In the synthesis process, we will examine the range of research questions across research traditions to compare and contrast the meta-narratives not only in terms of the results, but in how the issue has been conceptualized, theorized, and studied. Conflicting findings and contestations within and between traditions are treated as “higher order data and analysed interpretively to produce further insights” ([36], p. 10). Synthesis techniques involve multiple strategies, including [36]:

- Paradigm bridging—identifying commonalities in assumptions, concepts, theories, findings, and possibly philosophical underpinnings
- Paradigm bracketing—describing and explaining differences in the assumptions
- Interplay—exploring tensions in the data and explanations in the different meta-narratives
- Meta-theorizing—exploring and explaining patterns that cross different or conflicting understandings

The research team members will meet to discuss the key dimensions of the issues and will synthesize together each meta-narrative’s contributions to understanding the issues and answering the research questions. They will formulate preliminary conclusions [32] based on the synthesis. When the synthesis is completed, the full team will meet to reflect on, discuss, and interpret the findings. In particular, they will review the observed contestations with the goal of developing a robust explanation for the contradictory evidence. Preliminary recommendations for policy and public health practice will be developed during the team meeting. The evidence synthesis and preliminary recommendations will once again be presented by the knowledge users to their respective organizations for their feedback, which will be incorporated into the synthesis.

### Recommendation phase

This is the sixth and final phase of the review process. Through reflection, team dialogue, and feedback from other end users, key messages will be summarized. A final team meeting will draft final recommendations for practice, policy, and further research. After the draft is completed, the full team will identify a broad range of potential stakeholders and will develop knowledge translation strategies for disseminating findings with the assistance of the CARBC knowledge translation specialist. Some possible products include research bulletins, policy briefings, policy forums, and on-line information resources. The results and policy recommendations from the meta-narrative synthesis will also be communicated through traditional academic channels: conference presentations (in particular, the annual Canadian Public Health Association Conference) and academic journal articles (this protocol paper, a methodology paper, and at least one article on the findings). Reporting will be informed by the RAMESES publication standards [36].

## Discussion

The RAMESES Project has published a set of quality standards for meta-narrative reviews [48]. In this protocol, we strive to meet or exceed these standards. For the research questions, the topic is suitable for meta-narrative review because studies on vapour devices come from many different research traditions and the findings are contested. This methodology allows us to synthesize the diverse types of evidence that will answer our specific research questions with the explicit goal of resolving the conflicting conclusions within and between these traditions. Focusing the review will occur at multiple stages, not just the planning phase, through the feedback of the knowledge users, their organizations, and from additional interested stakeholders who have requested to be involved with this review. The search strategy includes a broad range of databases, hand searching, and grey literature retrieval, followed with citation chasing for seminal articles. Quality assessment tools developed for meta-narrative review have been selected, and additional appraisal tools may be added as we develop an understanding of the different standards in the research traditions we identify. Data extraction categories will be determined in the mapping phase and further refined during the appraisal phase. The synthesis phase has the expressed goal of synthesizing the evidence to answer the research questions and to review, explain, and potentially resolve the contestations that bedevil discussions on vapour devices. Planning for reporting and knowledge translation products are incorporated throughout the project, and will be refined with the input of knowledge users, their staff, and stakeholders. While it is impossible to foresee every process in this iterative methodology, our research design and diverse research team have the capacity to execute a meta-narrative synthesis that will enable the different traditions to “tell their story” on vapour devices.

Our meta-narrative review will offer a fuller picture of the literature on vapour devices than prior reviews. We have the broadest search strategy yet conducted because it retrieves papers using the full assortment of terms for vapour devices which was not the case in most other reviews. Furthermore, our search included more databases than prior reviews. The result will be a far greater inclusion of literature from diverse fields. In addition, our meta-narrative analysis incorporates the editorials and news items which make up the majority of publications on vapour devices, allowing us to expose and explain the debates occurring beyond the pages of research studies that influence the interpretation of the evidence garnered in the empirical studies. And the debate is heated between those who believe that vapour devices are a new hazard in the tobacco epidemic, and those who hope that vapour devices will provide a less harmful product for addicted smokers, or a potential cessation treatment. What public health policies and regulations will be appropriate responses for vapour devices? In this fraught environment, public health officials require a cool, comprehensive appraisal of conflicting evidence, a review that is urgently needed due to the rapid increase in sales of vapour devices. In our project, we seek to clear the air with a meta-narrative synthesis of the evidence and to provide recommendations for policy, regulation and future research.

## Additional files

Additional file 1: PRISMA-P 2015 checklist. Additional file 2: Search strategy.
