# Supplementary material for: Clearing the air: protocol for a systematic meta-narrative review on the harms and benefits of e-cigarettes and vapour devices
Source: Syst Rev. 2016 May 21;5:85. doi: 10.1186/s13643-016-0264-y (PMC4875675; doi:10.1186/s13643-016-0264-y)
Supplement: Additional file 1: — PRISMA-P 2015 checklist. [file 13643_2016_264_MOESM1_ESM.docx]

Additional File 1 MacDonald Clearing the Air

**PRISMA-P 2015 checklist**

| **ADMINISTRATIVE INFORMATION** | | |
| --- | --- | --- |
| **Title** item 1a | | |
| **Identification** |  | Clearing the Air: A systematic meta-narrative review on the harms and benefits of e-cigarettes and vapour devices, Protocol. |
| **Update** | Item 1b | This is an original review, not an update of a prior review. |
| **Registration** | Item 2 | PROSPERO CRD42015025267 |

**Authors** item 3a

Dr. Marjorie MacDonald, RN, PhD (corresponding author)

Professor, School of Nursing

University of Victoria

PO Box 1700 STN CSC

Victoria BC V8W 2Y2

[Marjorie@uvic.ca](mailto:Marjorie@uvic.ca)

Renée O’Leary, PhD(c)

Doctoral Candidate

Social Dimensions of Health

Graduate Student

Centre for Addictions Research of British Columbia

University of Victoria

PO Box 1700 STN CSC

Victoria BC V8W 2Y2

[kholeary@uvic.ca](mailto:kholeary@uvic.ca)

Dr. Tim Stockwell, PhD.

Director, Centre for Addictions Research of British Columbia

University of Victoria

PO Box 1700 STN CSC

Victoria BC V8W 2Y2

timstock@uvic.ca

Dan Reist

Assistant Director, Knowledge Exchange

Centre for Addictions Research of British Columbia

University of Victoria

PO Box 1700 STN CSC

Victoria BC V8W 2Y2

[dreist@uvic.ca](mailto:dreist@uvic.ca)

**Contributions** item 3b

MM is the guarantor of the review. MM and RO contributed equally to designing the systematic review and drafting the protocol. RO developed the search strategy. DR provided substantial contribution to the knowledge translation components of the review and overall feedback on the project. TS approved the study design, and provided feedback on the protocol draft. All authors have read and approved the final manuscript.

| **Amendments** | Item 4 | The Research Coordinator (RO) will document and report any amendments to the protocol. |
| --- | --- | --- |
| **Support** | | |
| **Sources** | Item 5a | The review is funded by the Canadian Institutes for Health Research Knowledge Synthesis Grant, Funding Reference Number 138211. The Centre for Addictions Research of British Columbia is providing office space, administrative support, and teleconference facilities. |
| **Sponsor** | Item 5b | Canadian Institutes for Health Research |
| **Role of sponsor/funder** | 5c | The Funder had no role in the development of the protocol. |

| **INTRODUCTION** | |
| --- | --- |
| **Rationale** Item 6  The sale and use of e-cigarettes and other vapour devices is increasing dramatically, and a contentious debate has risen within public health over the harms and benefits of these devices. *Clearing the Air* seeks to clarify the issues with a systematic review that informs the pressing regulatory and public health decisions to be made regarding these new products.  **Objectives** Item 7  Our research questions are: |  |
| 1. What are the health risks and benefits of vapour devices, and how do these compare to cigarettes? 2. What is the harm reduction potential of vapour devices for individuals, the environment, and society? 3. Does youth vapour device experimentation lead to cigarette use? 4. Can vapour devices be effective tools for tobacco cessation? 5. What is the potential toxicity of second-hand vapour? |  |
| **METHODS**  **Eligibility criteria** Item 8  Eligibility: all published academic articles on or discussing vapour devices, and publications on vapour devices from health and medical NGOs, including non-English publication. The publications will include articles, letters, editorials, conference abstracts, and poster presentations. Thesis and dissertations are excluded.  **Information sources** Item 9  The search period is 2007 – October 2, 2015. The first academic article on vapour devices was published in 2007.  The search includes 15 academic databases:  Academic Search Complete (EBSCO)  Business Source Complete (EBSCO)  CINAHL with full text (EBSCO)  Cochrane Central Register of Controlled Trials (CENTRAL) (Ovid)  LGBT Life with full text(EBSCO)  LILACS (Latin American and Caribbean Literature on Health Sciences)  MEDLINE (Ovid) (second search)  PapersFirst (OCLC)  ProceedingsFirst (OCLC)  PsycARTICLES (EBSCO)  PubMed  ScienceDirect (Elsevier)  Web of Science (Science Citation Index, the Social Sciences Citation Index) (ISI)  Women’s Studies International (EBSCO)  WorldCat (OCLC)  The first 300 entries in Google Scholar.  The following journals will be hand searched:  Tobacco Control, Nicotine and Tobacco Research, Tobacco Journal International (trade journal), Tobacco Induced Diseases.  Grey literature is being retrieved from the following list of NGOs:  Canadian Public Health Association  Heart and Stroke Foundation  Canadian Cancer Society  Canadian Lung Association  Canadian Convenience Stores Association  NSRA – Non-Smokers Rights Association  Ontario Tobacco Research Unit – RECIG  Physicians for a Smoke-Free Canada  Tobacco Harm Reduction Association of Canada (vaper organization)  Consumer Advocates for Smoke-Free Alternatives (vaper organization)  Electronic Cigarette Trade Association of Canada  US Department of Health and Human Services  US Centers for Disease Control and Prevention  US Food and Drug Administration  UK National Institutes for Clinical Excellence  UK Royal College of Physicians  UK Centre for Tobacco and Alcohol Studies  Harm Reduction International  Nicotine Science and Policy  Tobacco Tactics  Stanton Glantz blog  Michael Siegel blog  Ecigarette Research blog  **Search strategy** Item 10  ScienceDirect (Elsevier)  Subject Headings/Search Terms: electronic cigarette*, “electronic nicotine”, vaping  Fields searched: abstract, title, keywords  Date parameters: 2007-October 2, 2015 (present)  **Study records**  **Data management** Item 11a  Reference management with EndNote. Publications PDFs downloaded into NVivo.  **Selection process** Item 11b  The initial search publications will be mapped into topic areas by the Research Coordinator (RO) to determine the main concepts, theories, methods, and questions that characterize each research tradition. The publications will be given an overall quality assessment by the Greenhalgh et al. quality criteria, and will be rated as outstanding, having some limitations, or having many limitations, and will be additionally rated as essential, included, or of marginal relevance. Citation metrics will be obtained for articles that have been published a minimum of 6 months. Based on these initial criteria, all studies brought forward will be assessed for quality within the relevant research tradition, with conflicts resolved by the principle investigator (MM).  **Data collection process** Item 11c  Because meta-narrative review is an interpretive and constructivist process about making sense of a body of literature rather than a technical process of categorizing data according to a checklist, we will select and combine data judiciously from primary sources. Data extraction sheets will be developed inductively.  **Data items** Item12  Data categories are anticipated to include main concepts, theories, methods, research questions, and evidence.  **Outcomes and prioritization** Item 13  Publications addressing any of the research questions will be mapped by research tradition, with particular attention to contestations, conflicting findings, and discordant conclusions.  **Risk of bias in individual studies** Item 14  Quality assessment checklists from Greenhalgh will be noted for each included publication. For meta-narrative synthesis, bias is a factor of study quality that does not change the publication’s contribution to the narrative of the research tradition.  **Data synthesis**  Items 15 a, b, c No quantitative synthesis is planned.  Item 15 d  An initial summary of the studies will include the main concepts, theories, methods, questions, and instruments that characterize each research tradition as well as the empirical findings, discrepant conclusions, overall strengths and limitations, and the contributions to the research question offered by each research tradition. For the final synthesis we may not be able to develop a single conceptual framework because of the diversity of research questions, methods, and findings. However, we will create multiple syntheses within and across traditions, synthesizing at higher levels of abstraction to the extent possible. Synthesis techniques will include paradigm bridging, paradigm bracketing, interplay, and meta-theorizing – accepted techniques within meta-narrative review methodology.  **Meta-bias(es)** Item 16  Individual research traditions will be examined for selective reporting and conflicting data. Non-English studies will be summarized in English and be eligible for inclusion as per the selection criteria. Historical context is a key component of meta-narrative analysis, and will be reported as part of the synthesis.  **Confidence in cumulative evidence** Item 17  The strength of the synthesis will be evaluated with the Greenhalgh system. |  |
|  |  |
|  |  |
|  |  |
|  |  |
|  |  |
