# Supplementary material for: Clearing the air: protocol for a systematic meta-narrative review on the harms and benefits of e-cigarettes and vapour devices
Source: Syst Rev. 2016 May 21;5:85. doi: 10.1186/s13643-016-0264-y (PMC4875675; doi:10.1186/s13643-016-0264-y)
Supplement: Additional file 2: — Search strategy. [file 13643_2016_264_MOESM2_ESM.docx]

Additional File 2

**Search Strategy**

Primary Search Terms: “electronic cigarette”, e-cigarette, “electronic nicotine”, vaping

Secondary Search Terms: e-hookah, e-cigar, e-vapor, e-liquid, vape pen, vapers, vape shops

The Research Coordinator will keep a record of all searches, changes made to searches, and dates searches were executed.

Search fields: subject headings, keywords, title, and abstract.

Search period: 2007 – April 1, 2015

Start date based on the date of first publication on electronic cigarettes.

Databases:

Academic Search Complete (EBSCO)

Business Source Complete (EBSCO)

CINAHL with full text (EBSCO)

Cochrane Central Register of Controlled Trials (CENTRAL) (Ovid)

LGBT Life with full text(EBSCO)

LILACS (Latin American and Caribbean Literature on Health Sciences)

MEDLINE (Ovid) (second search)

PapersFirst (OCLC)

ProceedingsFirst (OCLC)

PsycARTICLES (EBSCO)

PubMed

ScienceDirect (Elsevier)

Web of Science (Science Citation Index, the Social Sciences Citation Index) (ISI)

Women’s Studies International (EBSCO)

WorldCat (OCLC)

Google Scholar first 300 entries

Hand Search Journals: Tobacco Control, Nicotine and Tobacco Research, Tobacco Journal International (trade journal), Tobacco Induced Diseases

Publications included: academic articles, letters, editorials, conference abstracts, poster presentations

Non-English publications included.

Publications excluded: thesis and dissertation

**Grey Literature Search**

Canadian Public Health Association

Heart and Stroke Foundation

Canadian Cancer Society

Canadian Lung Association

Canadian Convenience Stores Association

NSRA – Non-Smokers Rights Association

Ontario Tobacco Research Unit – RECIG

Physicians for a Smoke-Free Canada

Tobacco Harm Reduction Association of Canada (vaper organization)

Consumer Advocates for Smoke-Free Alternatives (vaper organization)

Electronic Cigarette Trade Association of Canada

US Department of Health and Human Services

US Centers for Disease Control and Prevention

US Food and Drug Administration

UK National Institutes for Clinical Excellence

UK Royal College of Physicians

UK Centre for Tobacco and Alcohol Studies

Harm Reduction International

Nicotine Science and Policy

Tobacco Tactics

Stanton Glantz blog

Michael Siegel blog

Ecigarette Research blog
